# Population genetics of ectoparasitic mites suggest arms race with honeybee hosts

**DOI:** 10.1038/s41598-019-47801-5

**Published:** 2019-08-06

**Authors:** Alexis L. Beaurepaire, Arrigo Moro, Fanny Mondet, Yves Le Conte, Peter Neumann, Barbara Locke

**Affiliations:** 1 INRA, UR 406 Abeilles et Environnement, Avignon, France; 2 Vetsuisse Faculty/University of Bern, Institute of Bee Health, Bern, Switzerland; 3 Agroscope, Swiss Bee Research Center, Bern, Switzerland; 4 Department of Ecology, Swedish University of Agricultural Sciences, Uppsala, Sweden

**Keywords:** Evolutionary genetics, Population genetics, Entomology, Molecular ecology, Evolutionary genetics

## Abstract

The ectoparasitic mite, *Varroa destructor*, is the most severe biotic threat to honeybees (*Apis mellifera*) globally, usually causing colony death within a few years without treatments. While it is known that a few *A. mellifera* populations survive mite infestations by means of natural selection, the possible role of mite adaptations remains unclear. To investigate potential changes in mite populations in response to host adaptations, the genetic structure of *V. destructor* in the mite-resistant *A. mellifera* population on Gotland, Sweden, was studied. Spatio-temporal genetic changes were assessed by comparing mites collected in these colonies, as well as from neighboring mite-susceptible colonies, in historic (2009) and current (2017/2018) samples. The results show significant changes in the genetic structure of the mite populations during the time frame of this study. These changes were more pronounced in the *V. destructor* population infesting the mite-resistant honeybee colonies than in the mite-susceptible colonies. These results suggest that *V. destructor* populations are reciprocating, in a coevolutionary arms race, to the selection pressure induced by their honeybee host. Our data reveal exciting new insights into host-parasite interactions between *A. mellifera* and its major parasite.

## Introduction

Anthropogenic movement of species can threaten biodiversity, agriculture, ecosystem functioning and can facilitate the spread of harmful pathogens^1,2^. The western honeybee, *Apis mellifera*, is a perfect example of a species that has experienced a rapid expansion in geographical distribution due to international trade and globalization and in this process has aquired many novel parasites and pathogens^3–5^.

The most dramatic consequence of the global spread of *A. mellifera* is the propagation of the invasive ectoparasitic mite, *Varroa destructor*. This mite is inarguably the most severe threat to *A. mellifera* globally, practically exterminating wild colonies and severly affecting the management and profitability of beekeeping in the wake of its global spread during the 1980’s and 1990’s^6^. The damage this parasite causes to its new host by feeding on adults and brood is amplified by the multiple viruses it carries and transmits^7–9^.

*V. destructor*, originally restricted to the Asian continent, has a non-lethal relationship with its natural host, the eastern honey bee, *Apis cerana*^10^. However, due to the transportation and introduction of *A. mellifera* in Asia, the mite managed to switch host and has successfully established itself as a harmful parasite in *A. mellifera* honeybee colonies^11^. Without a long-term coadaptive evolution, as is shared between the mite and its natural host, *A. mellifera* colonies are ill-equiped to cope with this new invasive parasite.

Current treatment strategies used in apiculture for the control of *V. destructor* infestations are costly, time-consuming, can harm the host and alter the quality of bee products^6^. Despite these drawbacks, most colonies of *A. mellifera* require treatment to have a chance for surviving the parasite infestation for more than 1–2 years. Yet, a few populations of *A. mellifera* exhibit resistance or tolerance traits that allow them to survive extended periods without treatments^12^.

To understand how these colonies deal with the invasive parasite, surviving honeybee populations have been studied extensively in the past decades. This research has highlighted that a wide range of individual or colony-level mechanisms are involved in their survival and that many of these traits are inheritable^13–17^. One such well-studied isolated honeybee population on the island of Gotland, Sweden, has been living treatment-free for almost two decades^12^. This population was established in the late 1990’s as an isolated natural selection experiment, allowing *A. mellifera* bees to naturally adapt to *V. destructor* parasitism^18^. This experiment resulted relatively quickly in improved survival rates through naturally adapted traits that limit the reproductive success of *V. destructor* and are genetically inheritable within the population^16,18,19^.

Host-parasite coevolutionary theory predicts that adapted host resistance traits, in response to parasitic pressure, are expected to induce a reciprocated selection pressure on the parasite, strong enough to drive counter adaptations towards a fitness optima^20,21^. This ultimately results in an arms race with a series of adaptations and counter-adaptations between the host and the parasite^22^. In most senarios, the parasite, usually with a shorter generation time than its host, would have an advantage in this arms race^23,24^. However, in this particular system, *V. destructor* is at a disadvantage due to an important factor: the invasive mite population in Europe has a low level of genetic diversity^25^. This is in part due to a founder effect abolishing most genetic diversity during only a few invasion occasions from its original host, but also due to the reproductive biology of the mite with frequent incestuous mating^25^. Within the sealed worker brood cells of developing *A. mellifera* pupae, the mother mite produces a single haploid male offspring followed by 4–5 diploid females^26^. The adult male copulates multiple times with its adult sisters before the parasitized bee emerges as an adult. At which point, the male dies in the cell and the fertilized daughters along with their mother will enter the honeybee colony’s mite population growth cycle with on average 10–15 generations per year^6^. This incestuous reproductive system generates high inbreeding levels. Nevertheless, occasionally two mites may enter a brood cell together and mating can then occur between lineages introducing genetic admixing^27^.

To date, it is unclear if or how mites reciprocate with antagonistic adaptations to their adapted host. Antagonistic coevolution (such as that between hosts and parasites) is expected to drive molecular evolution leading to genetic divergence between populations at a much faster rate compared to selection pressures of environmental change^28,29^. The aim of this study was therefore to analyse temporal genetic structure changes of *V. destructor* mites infesting the naturally adapted mite-resistant honeybee population on Gotland, Sweden, to investigate whether the parasite population shows signs of reciprocated adaptations in response to the resistant adaptations of its host. The mites from the mite-resistant population on Gotland were compared with a geographically neighboring mite population from managed mite-susceptible honeybees that have not experienced natural selection pressures. The changes in the genetic diversity and genetic structure was compared over time using microsatellites to compare historic mite samples collected in 2009 with current mite samples collected from the same populations in 2017 and 2018.

## Results

A total of 432 *V. destructor* adult females were genotyped at nine polymorphic microsatellite markers to study the temporal genetic structure of the mites in the mite-resistant colonies and local susceptible colonies from 2009 (“historic samples”) and 2017–18 (“current samples”) located on the island of Gotland, Sweden (Table 1). In addition, we used samples from the mainland from the apiary of the University of Uppsala to control the degree of isolation of the Gotland mite populations. Overall, a low number of alleles was found for all markers (N_A_ = 2–3, Table S1). An analysis of linkage disequilibrium revealed that none of the marker pair was significantly linked after Bonferroni corrections for multiple testing (all *p*-values > 0.00035), confirming the independence of the markers used.

**Table 1:** Information on the samples.

Groups	Period	GPS coord.	N_Hives_	N_Ind_
Resistant	Historic 2009	57°4′7. 3″N 18°12′27. 0″E	8	146
Susceptible		57°8′9.4″N 18°18′46.0″E	11	108
Resistant	Current 2017–18	57°4′7. 3″N 18°12′27. 0″E	3	54
Susceptible		57°22′27. 0″N 18°40′24. 3″E	4	41
Mainland		59°49′4. 9″N 17°39′22. 9″E	4	83

Table indicating the group names (Groups), period of sampling (Period), GPS coordinates (GPS coord.), number of hives (N_Hives_) and number of individuals for each group (N_Ind_) used in this study.

The comparison of genetic diversity showed that the number of alleles and the level of heterozygosity did not differ significantly among the five *V. destructor* groups (Kruskall Wallis, *p* > 0.05) (Fig. S1). In addition, all five groups significantly deviated from the Hardy-Weinberg equilibrium (*p* < 0.001). Notably, a few private alleles were detected in all groups, but these alleles had a very low frequency (<3%). Finally, a rarefaction analysis to assess the impact of sample size on the estimates of genetic diversity showed that the level of allelic richness reaches a plateau after about 20 mites are included, irrespective of the group considered (Fig. S2).

**Figure 1:**
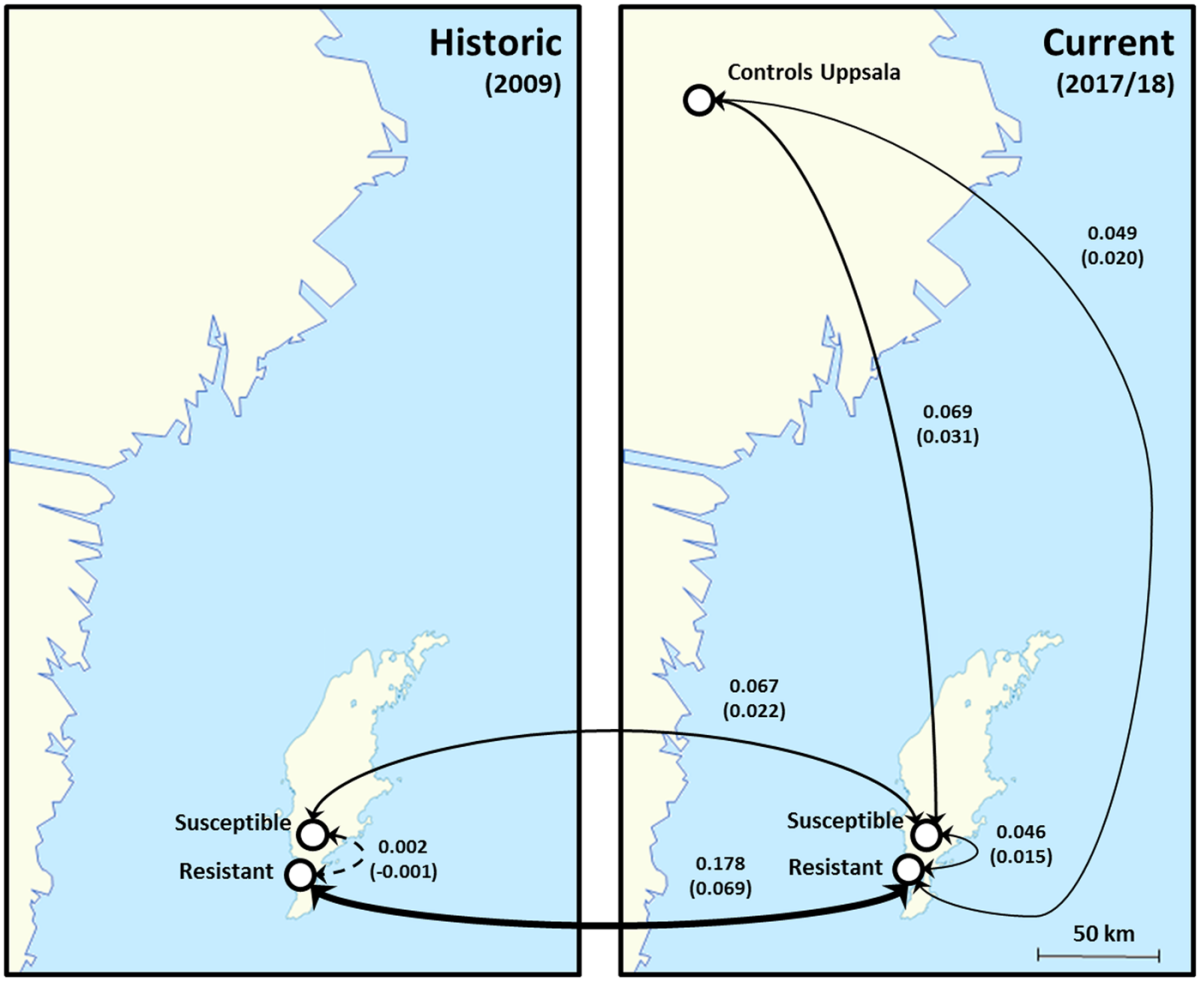
Genetic differentiation across populations of *Varroa destructor*. Schematic maps showing the results of the pairwise genetic differentiation among groups. Significant results are represented by full lines, non-significant by dashed lines, the size of the lines is proportional to the values of the estimates, also indicated next to the relevant lines (on top: F_ST_ values, and D_est_ below and between parentheses).

To assess the level of genetic differentiation between the mite groups, we calculated two distinct pairwise genetic differentiation indexes (F_ST_ and D_est_). These tests indicated different patterns between the groups compared (Table 2, Fig. 1). Minute and non-significant levels of genetic differentiation were found between mites coming from naturally-surviving and susceptible colonies of the historic collection (F_ST_ = 0.002 and D_est_ = 0.000, *p* = 0.720). In contrast, the level of differentiation between these two groups was more marked and significant in the current samples (F_ST_ = 0.046 and D_est_ = 0.015, *p* < *0.01*). In parallel, when comparing mites from the same host population between the two sampling dates, more elevated and significant levels of population differentiation were obtained. Notably, this difference was about three times higher in the samples from the resistant colonies (F_ST_ = 0.178 and D_est_ = 0.069, *p* < 0.001) compared to the susceptible ones (F_ST_ = 0.067 and D_est_ = 0.022, *p* < 0.01). Finally, the comparison between the samples from the mainland and the two current Gotland populations indicated low but highly significant genetic differentiation (Table 2, Fig. 1).

**Table 2:** Results of the pairwise analysis of genetic differentiation.

Groups		F_ST_	D_est_	*p*
S-historic	R-historic	0.002	0.000	0.720
S-current	R-current	0.046	0.015	<0.01
S-historic	S-current	0.067	0.022	<0.01
R-historic	R-current	0.178	0.069	<0.001
S-current	Mainland	0.069	0.031	<0.001
R-current	Mainland	0.049	0.020	<0.001

Table indicating the results of the pairwise population differentiation analysis, showing the groups compared (Groups), the level of genetic differentiation using two distinct estimates (F_ST_ and D_est_) and the associated *p*-values based on 9999 permutations.

**Figure 2:**
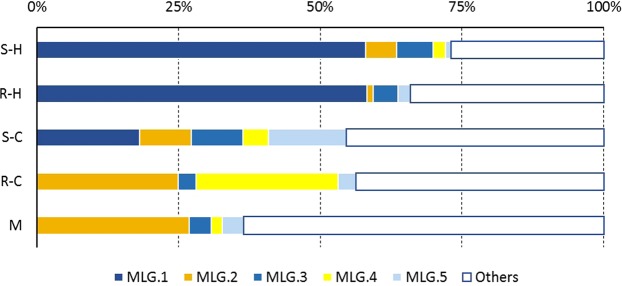
Distribution of the most prevalent MLGs across groups. Prevalence of the different *V. destructor* Multi-Locus Genotypes (MLGs) across the different groups. The coloured bars represent the five most prevalent MLGs and the white bars represent the rest of the MLGs. The letters represent the populations (S: Susceptible, R: resistant, M: mainland) and dates (H: historic and C: current).

To investigate how individual mite genotypes were represented in the five groups, we analysed the frequency and the distribution of Multi-Locus Genotypes (MLGs) in our samples. Overall, 64 MLGs were detected (Table 3). The mainland group was the most diverse, with 27 MLGs detected and 19 private MLGs (i.e. MLGs sampled only in one group). In Gotland, the number of MLGs found in the historic samples was higher (N = 33) than in the current samples (N = 22). However, the number of private MLGs was higher in the current samples from the mite-resistant group, with eight unique MLGs accounting for 34.38% of the mites of this group. The comparison of the distribution of the five most prevalent MLGs across populations and periods revealed highly significant differences across groups (Chi² test, p < 0.001). Interestingly, the most predominant MLG in the historical samples (>58% in both populations) was not found in the current mite-resistant group but was found in the current susceptible group (18.18%) (Fig. 2).

**Table 3:** Distribution and prevalence of Multi-Locus Genotypes (MLGs).

Category		N	% of individuals
Number of Samples	S-historic	93	32.07%
	R-historic	91	31.38%
	S-current	22	7.59%
	R-current	32	11.03%
	Mainland	52	17.93%
	**Total**	290	100.00%
Number of MLGs	S-historic	19	32.07%
	R-historic	23	31.38%
	S-current	12	7.59%
	R-current	15	11.03%
	Mainland	27	17.93%
	**Total**	45	100.00%
	Historic	33	63.45%
	Current	22	18.62%
	**Total**	55	82.07%
	Susceptible	24	39.66%
	Resistant	34	42.41%
	**Total**	55	82.07%
Private MLGs	S-historic	8	10.75%
	R-historic	11	21.98%
	S-current	3	18.18%
	R-current	8	34.38%
	Island-current	15	44.44%
	Mainland	19	50.00%
	**Total**	32	20.17%
Distribution of the most prevalent MLG	S-historic	54	58.06%
	R-historic	53	58.24%
	S-current	4	18.18%
	R-current	0	0.00%
	Mainland	0	0.00%
	**Total**	111	38.28%

The number (N) and % of individuals of the different categories indicated in the left column.

**Figure 3:**
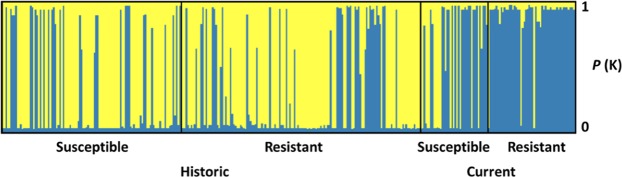
Genetic Structure of the Gotland mite populations. The blue and yellow colours indicate the two genetic clusters, the X-axis represents individuals and the Y-axis represents the probability to belong to the two different clusters (ΔK_2_ = 269.46).

Finally, to assess the spatio-temporal genetic changes in the four mite groups from the island of Gotland, an analysis of population structure was conducted using the software Instruct^30^. Here, the samples from the mainland were excluded to focus on the changes due to the host colony phenotype (resistant vs susceptible) and exclude any background noise from a potential island effect. This analysis revealed that the most likely number of genetic clusters in this mite sample was two (ΔK_2_ = 269.46). Displaying the individuals according to these two clusters (Fig. 3) revealed that most mites from the two historic groups belonged to the first cluster whereas samples from the two current groups were mostly of the second cluster. Accordingly, the likelihood of the four groups to be affiliated with the two genetic clusters was significantly different (χ² = 142.40, p < 0.001). In addition, when comparing the mites collected in the mite-resistant and the mite-susceptible colonies, significant differences were found in the current samples (χ² = 9.22, p < 0.01) but not in the historic ones (χ² = 0.25, p > 0.05).

## Discussion

The results of this study show that the genetic structure of *V. destructor* populations in the island of Gotland changed significantly between the time when our historic and current samples were collected, with differing degrees between the resistant and susceptible colonies. In addition, the distribution of the mite genotypes sampled in 2017 in the colonies from the resistant population suggests that genetic divergence in the parasite population of Gotland is ongoing as a result of selection by the host.

Despite marked differences in the number of individuals screened between the historic and the current groups, the markers used were able to accurately grasp the overall diversity in the populations studied here. For instance, when comparing the level of genetic diversity between the five *V. destructor* groups, no differences were found in the number of alleles or in the level of heterozygosity. Moreover, the rarefaction analysis performed on the five groups of mites did not reveal any difference between the groups with more samples (i.e. the historic groups) and the groups with less (i.e. the current groups), with a plateau phase being reached after about 20 individuals are analysed. This result is in line with the findings of another study using the same method^31^. Here, the lowest number of samples analysed in a group was well above this threshold (N = 41 in the S-current group). Additionally, populations of *V. destructor* are homogenous at the apiary level^31,32^. Therefore, clearly neither the difference in the number of colonies nor the difference in sample size used across the five groups had any significant impact on the genetic diversity estimates in our results.

Low levels of genetic diversity have initially been reported from a large number of *V. destructor* populations infesting *A. mellifera* throughout the world, suggesting that strong bottlenecks have taken place after this parasite’s host switch and further dispersal of the infested colonies^25^. Yet, more recent studies have revealed that the diversity of *V. destructor* is not as limited as previously thought in populations of Europe^27^, the USA^31,32^ and Asia^33,34^. The discrepancies between the earlier work on the varroa mite’s population genetics and the more recent investigations could be due to (*i*) the average low number of samples of the populations initially screened by Solignac *et al*. (2005) (avg = 12.44 mites per location), (*ii*) the degree of polymorphism of the markers screened, and/or (*iii*) an increased diversification of the parasite populations over the ten years separating the studies. Discriminating between the first two possibilities is not possible given the lack of investigations using the same markers as Solignac *et al*. (2005). Nevertheless, the results of this study suggest that the possibility (*iii*) is not a valid one, since we did not detect significant changes in the genetic diversity between our historic and current sample groups.

Despite this lack of differences in the level of genetic diversity over time, significant changes in population structure between the historic and current samples were detected. The low number and low frequency of private alleles detected demonstrate that these changes in population structure were mainly driven by variation in the frequency of the alleles initially found in the mite populations and not because of the addition or deletion of alleles. The results of the genetic structure analysis using the software Instruct did not show a pure segregation of the two genetic clusters across locations or dates, which can be explained by the fact that a unique lineage of *V. destructor* invaded *A. mellifera* colonies of Europe a few decades ago only^7,25,35^. Yet, despite this unique origin, significant genetic variation can now be observed, and the number of individuals belonging to the two genetic clusters detected here differed significantly across time and across the resistant and susceptible colonies in the current sampling period but not in the historic one. This temporal population structure was confirmed by the analysis of Multi-Locus Genotypes. The latter analysis permitted to assess the diversity and structure of *V. destructor* populations in a subtler way. Moreover, the differences of magnitude between the two indexes of population differentiation (F_ST_ and D_est_) reported also reflect these changes.

Hence, these independent but complementary analyses clearly indicate that *V. destructor* populations have changed within the eight years separating the collection of the current and historical samples. These changes led to a higher level of differentiation and an increased diversity of mite genotypes in the resistant colonies compared to the susceptible ones. These variations of population structure and diversity may reflect host-parasite interactions between *A. mellifera* and *V. destrutor*, but could also be caused by an influx of foreign mite genotypes in the current samples, the development of resistance to acaricide treatments and/or genetic drift. First, the comparison of the mainland mite population with the two populations located on Gotland suggest that an influx of mites was not responsible for the differences detected. Despite the common origin of the mites infesting both locations, the significant barrier to gene flow observed between the island and the mainland and the high proportion of private MLGs in the mainland and island mites in the current sampling period (50% and 44.44%, respectively) indicate that these populations are isolated. Second, both susceptible populations (in Gotland and Uppsala) are treated with oxalic acid, a compound that does not select specific resistant mites^36^. Thus, the changes of genetic structure detected is not likely be caused by the treatment of the susceptible colonies. Lastly, genetic drift, a process causing the allele frequencies of populations to vary from one generation to the next as a result of chance^37^, is probably not responsible for the integrality of the changes in population structure we observed within the time frame of our study. With a strong genetic drift effect, a reduction of diversity is expected^37,38^. In this study, there was no significant difference in the number of alleles and heterozysogity levels between the different mite populations from Gotland and from the mainland. In contrast, we found an increased diversity of MLGs in the resistant colonies of Gotland between 2009 and 2018. Moreover, if genetic drift was responsible for the increased differentiation between the two sampling dates, the most prevalent MLGs in the historical sampling period would be more likely to prevail in the current period^38^. Instead, the patterns of changes of mite MLGs between the two dates show that the most common MLG, representing more than half the mites sampled during the historical collection, was not sampled in the resistant group later on, but was still infesting the susceptible population. This observation suggests that a strong selection against the most common mite genotypes is occurring in the resistant colonies. To conclude, the above-mentioned evidences strongly suggest that the main factor responsible for the temporal changes in the genetic structure of *V. destructor* measured here are caused by the strong selection pressures induced through the co-evolution of the mite with its host, rapidly leading to observable genetic changes in the parasite population, potentially aiding their survival in adapted mite-resistant *A. mellifera* colonies.

The notion of *V. destructor* adaptation in response to its host or environment is not far fetched. Examples such as the ability of the mite to adapt to acaridice treatments^27,39^ show how quickly this parasite’s populations can react to selection pressures, despite it’s apparent lack of genetic diversity. In our study, given an average of ten generations of mites per year in the Northern-European climate of Gotland^40^, the time separating the collection of samples from the historic and current group is more than enough to assess the impact of strong selective forces guided by antagonistic coevolution^28,29^. *V. destructor* genetic variation has been linked to phenotypic variation associated with fertility and virulence differing between the two parasite haplotypes that spread globally from Japan and Korea^35,41^. When *V. destructor* was first reported in Brazil, the fertility and the virulence of the parasite were low, causing little damage to *A. mellifera* colonies^42^. However, only a few years later, the virulence of  *V. destructor* had increased in the region similar to what was experienced in European populations at the time. Genetic analyses demonstrated that the initial avirulent Japanese *V. destructor* haplotype had been replaced by the more virulent Korean haplotype^43^. The fact that variability in the consequences of mite infestation so clearly exists within the *V. destructor* haplotypes, having a significant effect on colony survival, suggests that disregarding the possibility of, for example, reduced virulence adaptions of the mite influencing host survival, is no longer reasonable. Indeed reduced virulence in mites has been proposed previously to explain the long term survival of another isolated honeybee population in the Arnot Forest in Ithaca, NY^44^.

In 2006, Fries and Bommarco performed a cross-infection experiment with the Gotland resistant honeybee population to test for varying host responses to mites, sourced from either the resistant or susceptible populations^45^. In their study, only 3 years before the historical samples of this study were taken, they found that mite source had no effect on the colonies and that Gotland colonies had significantly reduced mite infestation rates. Since their study was published, there has been a consensus in the scientific community to regard the mite as a “fixed factor” in the studies of underlying mechanisms to explain the long-term survival of naturally adapted honeybee populations, supported by the limited genetic diversity in the mite population^25^. The historic mite samples of this study confirm the results of Fries and Bommarco’s in 2006 that the mite source did not influence the bees, since the mites in 2009, only 3 years later, did not significantly differ genetically between groups. However, by looking over a decade after Fries and Bommarco’s study it is evident that a change has occurred in the genetic structure of the mite populations between surviving and treated colonies. Performing a similar cross-infection experiment testing the current mite genotype in the Gotland resistant and susceptible colonies will show whether the genotypic differences observed in the current samples of this investigation are associated with phenotypic differences and will help understanding how the diversity of *V. destructor* populations observed at the genetic level may impact the survivability of their hosts. If the genetic changes on the neutral markers of this study are confirmed by phenotypic changes, the recently updated version of the genome of the mite^46^ could be used to unravel the genetic bases of the parasite adaptations. Inhibiting mite reproductive success is a well defined genetically inheritable trait of the Gotland mite-resistant honeybee population, even if the mechanisms explaining how the bees are capable of this are still not completely clear^16^. Whether this host trait could explain the differences in mite genotype distribution we observed here remains to be studied.

In conclusion, the observed changes over time in the genetic structure of *V. destructor* suggest adaptations of the parasite, in a host-parasite coevolutionary arms race, most likely in response to selection pressures applied by the adapted resistant traits of the host. The magnitude of these changes between the historic and current samples of this study demonstrate a relatively fast response. Model systems for experimental host-parasite coevolution research has been largely restricted to microbes and short generation hosts^47^. The natural selection experiment on Gotland has been, and continues to provide, a unique oportunity to study host-parasite coevolution in real time between two larger eukaryote organisms.

## Material and Methods

### Sampling

In summer 2009, *V. destructor* adult females were collected from the brood of eight honeybee colonies in the naturally adapted mite-resistant honeybee population on the island of Gotland Sweden as well as from eleven mite-susceptible colonies of *A. mellifera *that require regular mite-treatement with oxalic acid to survive, located approximately ten km away from the resistant colonies (Table 1, Fig. 1). In the summer of 2017, mites were collected again from three colonies of the same population of mite-resistant *A. mellfera* colonies. However, control mites were collected in 2018, because parasite loads in treated colonies were very low in 2017 (most likely due to the regular treatments applied by beekeepers). In addition to the samples collected on Gotland, mites were collected on the mainland in Sweden from four mite-susceptible honeybee colonies at the Swedish University of Agricultural Sciences in Uppsala during the summer of 2017 as a secondary control group to account for a possible island effect. All mite samples were kept at −20 °C until DNA extraction. From hereinafter, the 2017 and 2018 samples will be refered to as the temporally “current” samples while the 2009 samples are refered to as the “historic” samples for streamlining the presentation and discussion of the data (Table 1).

### Genotyping

Total mite DNA was extracted using a Chelex protocol^48^. Mites were genotyped using a set of microsatellite markers. In all, 20 markers were tested to determine optimal PCR conditions (ie multiplexing) and polymorphism on a subset of 24 individuals from three colonies of the susceptible historic group. From these 20 markers, nine were polymorphic and worked well under our lab conditions (Table S1). PCR products were sent to Genoscreen (Lille, France) to run on a 3730XL sequencer (Applied Biosystems ®). Once received, the genotypes were scored manually using Peak Scanner v 1.0 (Applied Biosystems ®).

### Analyses

For the following analyses, the mite samples from Gotland were grouped according to their population of origin (resistant or susceptible) and the date of sampling (historic or current). The mite samples from Uppsala were included in the analyses and labeled as “mainland”. Only individuals with genotypes available for five or more loci were kept in this dataset. Along these lines, a total of 432 mites were kept in the analyses (Table 1).

First, the linkage disequilibrium between pairs of markers was tested along with the Hardy-Weinberg equilibrium for each group using Fstat v 2.9.3^49^. Then, to compare the genetic diversity across the five groups of mites, the number of alleles (N_A_) and the level of observed heterozygosity (Ho) were calculated using the Microsatellite Toolkit^50^ and compared using Kruskal-Wallis tests in R v. 3.5.2^51^. We used the software ADZE^52^ to conduct a rarefaction analysis on the allelic richness over markers for every mite groups to estimate whether the lowest sample size we used was sufficient to grasp accurately the genetic diversity of the group considered.

The software GenAlex v. 6.5^53^ was then used to assess genetic differentiation in the populations of *V. destructor* of in the four groups from Gotland and between the current mainland and Gotland samples by calculating two distinct estimates of population differentiation (F_ST_ and D_est_) as recommended by^54^. F_ST_ reflects the probability that two alleles drawn at random from within a group are identical by descent^55^ and is commonly used to compare the genetic differentiation between two or more group of samples^54,56^. Generally, F_ST_ values below 0.05 suggest low genetic differentiation across groups, whereas when this estimates ranges between 0.05 to 0.25, moderate genetic differentiation is suggested^37^. In parallel, D_est_ complements this estimate and better reflects differences in allele frequencies when markers are highly polymorphic^56,57^.

In addition, to explore the distribution of *V. destructor* genotypes, the package poppr^58^ for R v. 3.5.2^51^ was used to compare the prevalence of Multi-Locus Genotypes (MLGs) in the five mite groups. All individuals with missing data were excluded from this analysis (Table 3).

Finally, an analysis of genetic structure without *a priori* was performed using the software Instruct^30^ in order to infer the optimal number of genetic clusters underlying the mite sample (called “K”) from Gotland (parameters set as following: admixture model, 20 chains of each K varying from 1 to 8, 50’000 burn-in and 100’000 iterations). The methods from Evanno *et al*. (2005) was then used to determine the most likely K in the overall dataset. The results of the Instruct analysis were pooled using the software CLUMPP^60^ and a figure corresponding to the most likely number of K was prepared with the software Distruct^61^. In order to test whether the probabilities of belonging to a cluster differed among the different groups, a Chi-squared test was used to evaluate the relationship between the inferred clusters and the mite groups. To do so, the number of individuals in the different genetic clusters for each group was obtained from the ancestry values of the populations obtained with the CLUMPP software and was compared using a contingency table. In parallel, the differences between naturally-surviving and treated colonies for each sampling date was tested using the same method^59,62,63^.

## Supplementary information

Suppl. Info

## Data Availability

The datasets generated during and/or analysed during the current study are available from the corresponding author on reasonable request.
