# Supplementary material for: Population genetics of ectoparasitic mites suggest arms race with honeybee hosts
Source: Sci Rep. 2019 Aug 6;9:11355. doi: 10.1038/s41598-019-47801-5 (PMC6684582; doi:10.1038/s41598-019-47801-5)
Supplement: Supplementary file 1 — Suppl. Info [file 41598_2019_47801_MOESM1_ESM.docx]

**Population genetics of ectoparasitic mites suggest arms race with honeybee hosts**

Alexis L. Beaurepaire^1, *^, Arrigo Moro^2^, Fanny Mondet^1^, Yves le Conte^1^, Peter Neumann^2, 3^ and Barbara Locke^4^

1. INRA, UR 406 Abeilles et Environnement, Avignon, France
2. Vetsuisse Faculty/University of Bern, Institute of Bee Health, Bern, Switzerland
3. Agroscope, Swiss Bee Research Center, Bern, Switzerland
4. Department of Ecology, Swedish University of Agricultural Sciences, Uppsala, Sweden

* Correspondence to: alexis.beaurepaire@vetsuisse.unibe.ch

**Supplementary Information**


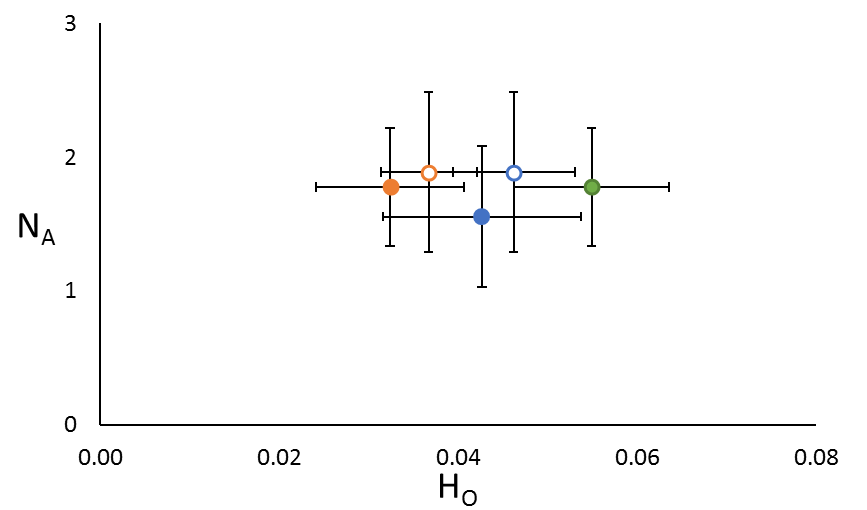


**Figure S1 – Comparison of the number of alleles (N_A_) and observed heterozygosity (H_O_)**

Mean levels of heterozygosity (X-axis) and mean number of alleles (NA) for the five groups studied are shown (green = mainland mite-suscpetible; orange = Gotland mite-resistant; blue = Gotland mite-susceptible; empty circles = historic samples; filled circles = current samples; Variance: SD).

**
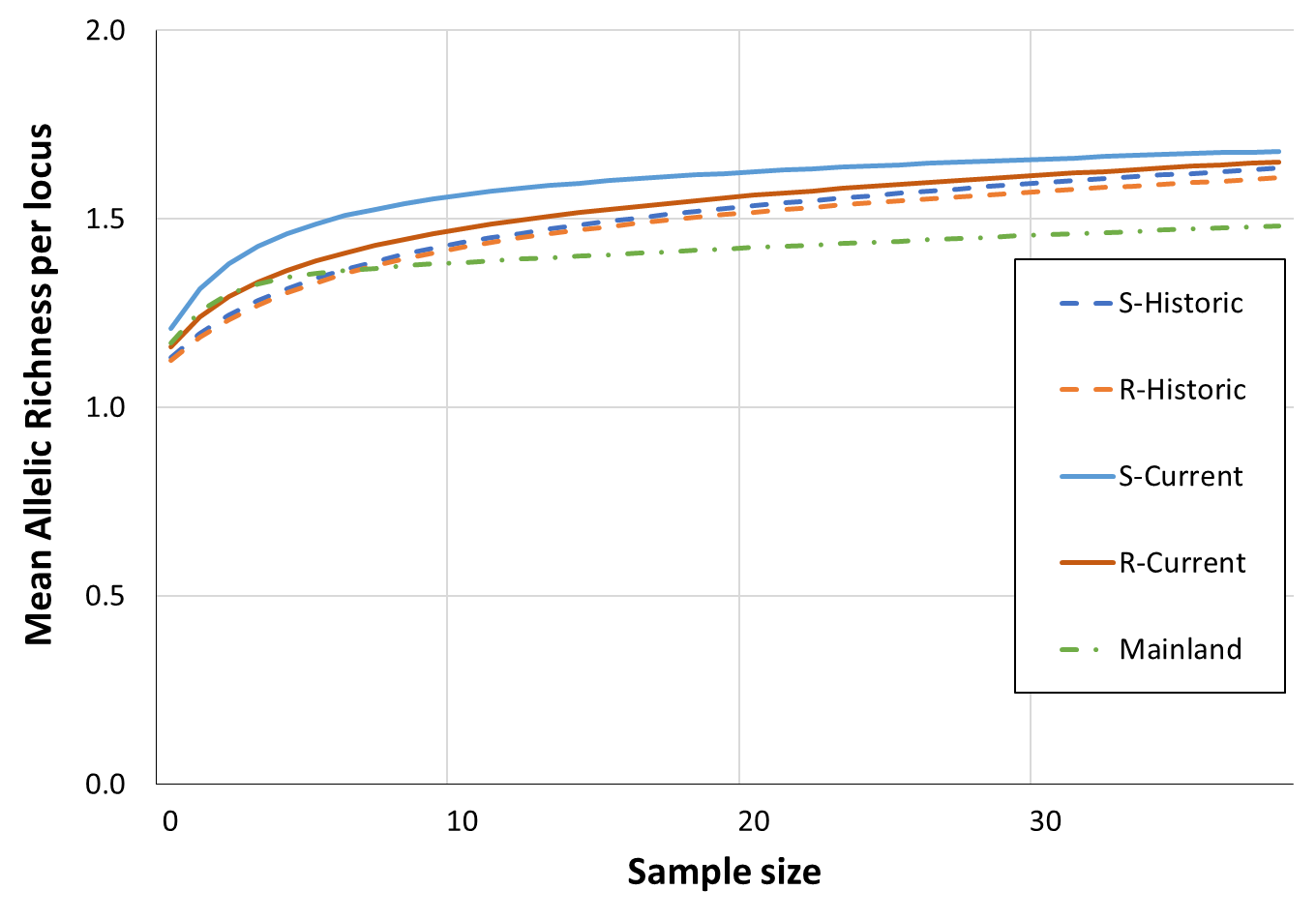
**

**Figure S2 – Rarefaction analysis**

Allelic richness in the five groups using a rarefaction approach estimated with the software ADZE ^52^.

**Table S1 – Information on the markers used in this study**

Table indicating how markers were used in multiplex or singly (Multiplexing), the name of the marker (Name), their reference (Ref), the average size of the marker products in base pairs (Size Avg) and the number of alleles (N_A_) found for each markers over all samples. References: 1: ^62^ 2: ^63^, 3: ^27^

| **Multiplexing** | **Name** | **Ref** | **Size Avg** | **N_A_** |
| --- | --- | --- | --- | --- |
| Group 1 | VD305 | 1 | 130 | 3 |
|  | VD307 | 1 | 160 | 2 |
| Group 2 | VJ294 | 2 | 170 | 2 |
|  | VJ295 | 2 | 155 | 3 |
| Group 3 | Vdes-01 | 3 | 400 | 2 |
|  | Vdes-03 | 3 | 300 | 2 |
| Group 4 | Vdes-02 | 3 | 300 | 2 |
|  | Vdes-04 | 3 | 270 | 2 |
| Single Marker | VJ292 | 2 | 230 | 3 |
